# Supplementary material for: Role and mechanism of NCAPD3 in promoting malignant behaviors in gastric cancer
Source: Front Pharmacol. 2024 Apr 22;15:1341039. doi: 10.3389/fphar.2024.1341039 (PMC11070777; doi:10.3389/fphar.2024.1341039)
Supplement: Supplementary file 11 [file DataSheet2.ZIP › GSEA/Transcipt factor/my_analysis.Gsea.1599461814734/DIDO1_TARGET_GENES.html]

Details for gene set DIDO1\_TARGET\_GENES[GSEA]

|  || Dataset | filtered\_dataset.sample\_info.cls#WT\_versus\_NCAPD3\_MUT |
| Phenotype | sample\_info.cls#WT\_versus\_NCAPD3\_MUT |
| Upregulated in class | WT |
| GeneSet | DIDO1\_TARGET\_GENES |
| Enrichment Score (ES) | 0.30706123 |
| Normalized Enrichment Score (NES) | 2.3063207 |
| Nominal p-value | 0.0 |
| FDR q-value | 0.04550819 |
| FWER p-Value | 0.056 |
Table: GSEA Results Summary

  

Fig 1: Enrichment plot: DIDO1\_TARGET\_GENES      
 Profile of the Running ES Score & Positions of GeneSet Members on the Rank Ordered List

  

| SYMBOL | TITLE | RANK IN GENE LIST | RANK METRIC SCORE | RUNNING ES | CORE ENRICHMENT || 1 | 55149 | MTPAP | 15 | 1.051 | 0.0018 | Yes |
| 2 | 7571 | ZNF23 | 25 | 1.018 | 0.0079 | Yes |
| 3 | 9759 | HDAC4 | 46 | 0.925 | 0.0041 | Yes |
| 4 | 376940 | ZC3H6 | 50 | 0.914 | 0.0136 | Yes |
| 5 | 91893 | FDXACB1 | 61 | 0.869 | 0.0170 | Yes |
| 6 | 117145 | THEM4 | 65 | 0.860 | 0.0258 | Yes |
| 7 | 157680 | VPS13B | 78 | 0.838 | 0.0272 | Yes |
| 8 | 9489 | PGS1 | 84 | 0.820 | 0.0339 | Yes |
| 9 | 55006 | TRMT61B | 101 | 0.782 | 0.0314 | Yes |
| 10 | 50861 | STMN3 | 126 | 0.777 | 0.0225 | Yes |
| 11 | 55687 | TRMU | 128 | 0.774 | 0.0318 | Yes |
| 12 | 7248 | TSC1 | 158 | 0.718 | 0.0182 | Yes |
| 13 | 817 | CAMK2D | 165 | 0.710 | 0.0226 | Yes |
| 14 | 10795 | ZNF268 | 168 | 0.710 | 0.0303 | Yes |
| 15 | 54971 | BANP | 172 | 0.707 | 0.0371 | Yes |
| 16 | 23590 | PDSS1 | 175 | 0.701 | 0.0446 | Yes |
| 17 | 57325 | CSRP2BP | 176 | 0.698 | 0.0537 | Yes |
| 18 | 51018 | RRP15 | 192 | 0.685 | 0.0507 | Yes |
| 19 | 84190 | METTL25 | 195 | 0.684 | 0.0580 | Yes |
| 20 | 9897 | KIAA0196 | 197 | 0.682 | 0.0661 | Yes |
| 21 | 55798 | METTL2B | 199 | 0.678 | 0.0741 | Yes |
| 22 | 145389 | SLC38A6 | 202 | 0.673 | 0.0812 | Yes |
| 23 | 7024 | TFCP2 | 204 | 0.671 | 0.0892 | Yes |
| 24 | 127253 | TYW3 | 225 | 0.654 | 0.0819 | Yes |
| 25 | 55599 | RNPC3 | 227 | 0.652 | 0.0895 | Yes |
| 26 | 84816 | RTN4IP1 | 232 | 0.649 | 0.0948 | Yes |
| 27 | 57521 | RPTOR | 239 | 0.645 | 0.0984 | Yes |
| 28 | 64794 | DDX31 | 248 | 0.633 | 0.1003 | Yes |
| 29 | 54865 | GPATCH4 | 261 | 0.622 | 0.0989 | Yes |
| 30 | 25974 | MMACHC | 265 | 0.621 | 0.1046 | Yes |
| 31 | 116143 | WDR92 | 267 | 0.619 | 0.1119 | Yes |
| 32 | 199857 | ALG14 | 275 | 0.615 | 0.1143 | Yes |
| 33 | 23341 | DNAJC16 | 276 | 0.613 | 0.1223 | Yes |
| 34 | 4238 | MFAP3 | 279 | 0.609 | 0.1286 | Yes |
| 35 | 84874 | ZNF514 | 283 | 0.606 | 0.1341 | Yes |
| 36 | 23036 | ZNF292 | 288 | 0.602 | 0.1388 | Yes |
| 37 | 54758 | KLHDC4 | 292 | 0.598 | 0.1442 | Yes |
| 38 | 81889 | FAHD1 | 299 | 0.592 | 0.1471 | Yes |
| 39 | 25852 | ARMC8 | 307 | 0.588 | 0.1492 | Yes |
| 40 | 129831 | RBM45 | 310 | 0.587 | 0.1553 | Yes |
| 41 | 84946 | LTV1 | 319 | 0.583 | 0.1565 | Yes |
| 42 | 27079 | RPUSD2 | 320 | 0.582 | 0.1641 | Yes |
| 43 | 6715 | SRD5A1 | 325 | 0.578 | 0.1684 | Yes |
| 44 | 55697 | VAC14 | 329 | 0.576 | 0.1735 | Yes |
| 45 | 94107 | TMEM203 | 333 | 0.574 | 0.1786 | Yes |
| 46 | 9617 | MTRF1 | 349 | 0.568 | 0.1741 | Yes |
| 47 | 2965 | GTF2H1 | 356 | 0.563 | 0.1767 | Yes |
| 48 | 5810 | RAD1 | 360 | 0.560 | 0.1816 | Yes |
| 49 | 10818 | FRS2 | 363 | 0.558 | 0.1873 | Yes |
| 50 | 57109 | REXO4 | 370 | 0.552 | 0.1897 | Yes |
| 51 | 115509 | ZNF689 | 374 | 0.551 | 0.1945 | Yes |
| 52 | 23361 | ZNF629 | 380 | 0.547 | 0.1976 | Yes |
| 53 | 60526 | LDAH | 403 | 0.535 | 0.1872 | Yes |
| 54 | 11019 | LIAS | 409 | 0.533 | 0.1902 | Yes |
| 55 | 147007 | TMEM199 | 410 | 0.533 | 0.1971 | Yes |
| 56 | 25917 | THUMPD3 | 412 | 0.530 | 0.2032 | Yes |
| 57 | 221786 | FAM200A | 417 | 0.525 | 0.2068 | Yes |
| 58 | 84274 | COQ5 | 419 | 0.523 | 0.2128 | Yes |
| 59 | 805 | CALM2 | 423 | 0.518 | 0.2172 | Yes |
| 60 | 91392 | ZNF502 | 433 | 0.513 | 0.2167 | Yes |
| 61 | 54931 | TRMT10C | 446 | 0.507 | 0.2138 | Yes |
| 62 | 148304 | C1orf74 | 447 | 0.506 | 0.2204 | Yes |
| 63 | 57478 | USP31 | 448 | 0.506 | 0.2270 | Yes |
| 64 | 8607 | RUVBL1 | 451 | 0.506 | 0.2320 | Yes |
| 65 | 27340 | UTP20 | 455 | 0.501 | 0.2361 | Yes |
| 66 | 200933 | FBXO45 | 456 | 0.500 | 0.2426 | Yes |
| 67 | 23383 | MAU2 | 459 | 0.500 | 0.2475 | Yes |
| 68 | 10785 | WDR4 | 465 | 0.495 | 0.2500 | Yes |
| 69 | 10969 | EBNA1BP2 | 483 | 0.485 | 0.2428 | Yes |
| 70 | 84261 | FBXW9 | 491 | 0.477 | 0.2435 | Yes |
| 71 | 29101 | SSU72 | 496 | 0.475 | 0.2465 | Yes |
| 72 | 11060 | WWP2 | 503 | 0.471 | 0.2479 | Yes |
| 73 | 54920 | DUS2 | 509 | 0.470 | 0.2500 | Yes |
| 74 | 5188 | GATB | 512 | 0.469 | 0.2545 | Yes |
| 75 | 130502 | TTC32 | 517 | 0.465 | 0.2574 | Yes |
| 76 | 90639 | COX19 | 535 | 0.454 | 0.2499 | Yes |
| 77 | 29090 | TIMM21 | 537 | 0.453 | 0.2550 | Yes |
| 78 | 55527 | FEM1A | 543 | 0.449 | 0.2569 | Yes |
| 79 | 1819 | DRG2 | 550 | 0.447 | 0.2579 | Yes |
| 80 | 79080 | CCDC86 | 555 | 0.443 | 0.2605 | Yes |
| 81 | 10773 | ZBTB6 | 562 | 0.441 | 0.2615 | Yes |
| 82 | 6632 | SNRPD1 | 563 | 0.440 | 0.2672 | Yes |
| 83 | 27349 | MCAT | 574 | 0.436 | 0.2650 | Yes |
| 84 | 57579 | FAM135A | 577 | 0.436 | 0.2691 | Yes |
| 85 | 196410 | METTL7B | 579 | 0.434 | 0.2739 | Yes |
| 86 | 55245 | UQCC1 | 580 | 0.434 | 0.2796 | Yes |
| 87 | 26057 | ANKRD17 | 589 | 0.431 | 0.2788 | Yes |
| 88 | 93134 | ZNF561 | 590 | 0.431 | 0.2844 | Yes |
| 89 | 55181 | SMG8 | 602 | 0.422 | 0.2812 | Yes |
| 90 | 6208 | RPS14 | 604 | 0.421 | 0.2859 | Yes |
| 91 | 7290 | HIRA | 612 | 0.417 | 0.2858 | Yes |
| 92 | 10445 | MCRS1 | 617 | 0.414 | 0.2880 | Yes |
| 93 | 22847 | ZNF507 | 618 | 0.413 | 0.2933 | Yes |
| 94 | 5147 | PDE6D | 624 | 0.410 | 0.2947 | Yes |
| 95 | 10061 | ABCF2 | 626 | 0.408 | 0.2992 | Yes |
| 96 | 26953 | RANBP6 | 630 | 0.406 | 0.3021 | Yes |
| 97 | 80110 | ZNF614 | 642 | 0.404 | 0.2987 | Yes |
| 98 | 79807 | GSTCD | 644 | 0.402 | 0.3031 | Yes |
| 99 | 57679 | ALS2 | 656 | 0.399 | 0.2996 | Yes |
| 100 | 78996 | C7orf49 | 661 | 0.396 | 0.3016 | Yes |
| 101 | 26747 | NUFIP1 | 666 | 0.394 | 0.3035 | Yes |
| 102 | 54476 | RNF216 | 672 | 0.389 | 0.3046 | Yes |
| 103 | 9831 | ZNF623 | 691 | 0.383 | 0.2954 | Yes |
| 104 | 285636 | C5orf51 | 692 | 0.382 | 0.3003 | Yes |
| 105 | 89910 | UBE3B | 693 | 0.381 | 0.3053 | Yes |
| 106 | 10607 | TBL3 | 698 | 0.380 | 0.3071 | Yes |
| 107 | 23076 | RRP1B | 709 | 0.370 | 0.3040 | No |
| 108 | 23350 | U2SURP | 719 | 0.364 | 0.3016 | No |
| 109 | 134430 | WDR36 | 733 | 0.359 | 0.2960 | No |
| 110 | 58509 | CACTIN | 748 | 0.351 | 0.2895 | No |
| 111 | 3188 | HNRNPH2 | 769 | 0.338 | 0.2780 | No |
| 112 | 55178 | RNMTL1 | 771 | 0.337 | 0.2816 | No |
| 113 | 54939 | COMMD4 | 777 | 0.335 | 0.2820 | No |
| 114 | 158 | ADSL | 796 | 0.326 | 0.2720 | No |
| 115 | 84522 | JAGN1 | 798 | 0.324 | 0.2754 | No |
| 116 | 51575 | ESF1 | 812 | 0.309 | 0.2692 | No |
| 117 | 5898 | RALA | 813 | 0.309 | 0.2732 | No |
| 118 | 4285 | MIPEP | 815 | 0.307 | 0.2764 | No |
| 119 | 9343 | EFTUD2 | 818 | 0.305 | 0.2788 | No |
| 120 | 26121 | PRPF31 | 825 | 0.301 | 0.2779 | No |
| 121 | 387680 | FAM21A | 828 | 0.297 | 0.2802 | No |
| 122 | 253725 | FAM21C | 829 | 0.297 | 0.2841 | No |
| 123 | 3146 | HMGB1 | 831 | 0.295 | 0.2871 | No |
| 124 | 23435 | TARDBP | 842 | 0.272 | 0.2827 | No |
| 125 | 1984 | EIF5A | 846 | 0.259 | 0.2837 | No |
| 126 | 23731 | TMEM245 | 891 | -0.306 | 0.2529 | No |
| 127 | 27230 | SERP1 | 892 | -0.306 | 0.2569 | No |
| 128 | 23412 | COMMD3 | 894 | -0.312 | 0.2602 | No |
| 129 | 7453 | WARS | 900 | -0.325 | 0.2604 | No |
| 130 | 84056 | KATNAL1 | 917 | -0.346 | 0.2523 | No |
| 131 | 3475 | IFRD1 | 935 | -0.360 | 0.2435 | No |
| 132 | 6397 | SEC14L1 | 941 | -0.364 | 0.2443 | No |
| 133 | 11077 | HSF2BP | 1008 | -0.408 | 0.1974 | No |
| 134 | 7871 | SLMAP | 1038 | -0.435 | 0.1801 | No |
| 135 | 80315 | CPEB4 | 1053 | -0.448 | 0.1749 | No |
| 136 | 5889 | RAD51C | 1059 | -0.451 | 0.1768 | No |
| 137 | 100534599 | ISY1-RAB43 | 1082 | -0.462 | 0.1654 | No |
| 138 | 5565 | PRKAB2 | 1094 | -0.474 | 0.1629 | No |
| 139 | 55370 | PPP4R1L | 1124 | -0.497 | 0.1464 | No |
| 140 | 22931 | RAB18 | 1231 | -0.608 | 0.0705 | No |
| 141 | 135114 | HINT3 | 1257 | -0.645 | 0.0591 | No |
| 142 | 8651 | SOCS1 | 1272 | -0.661 | 0.0566 | No |
| 143 | 5046 | PCSK6 | 1297 | -0.699 | 0.0467 | No |
| 144 | 23431 | AP4E1 | 1359 | -0.827 | 0.0093 | No |
| 145 | 8548 | BLZF1 | 1398 | -1.103 | -0.0065 | No |
| 146 | 493 | ATP2B4 | 1401 | -1.166 | 0.0071 | No |
Table: GSEA details [plain text format]

  

Fig 2: DIDO1\_TARGET\_GENES      
 Blue-Pink O' Gram in the Space of the Analyzed GeneSet

  

Fig 3: DIDO1\_TARGET\_GENES: Random ES distribution      
 Gene set null distribution of ES for **DIDO1\_TARGET\_GENES**
